# Synergistic effects of platelet-rich fibrin and CTLA4Ig gene-transfected porcine skin on accelerating wound healing in a rat model of deep second-degree burns: a mechanistic study

**DOI:** 10.3389/fimmu.2025.1756818

**Published:** 2026-01-19

**Authors:** Jiliang Li, Chongwei Xu, Leyi Chen, Jiaqi Lou, Hong Kong, Youfen Fan

**Affiliations:** 1 Burn Department, Ningbo No. 2 Hospital, Ningbo, Zhejiang, China; 2 Department of General Surgery, The Hospital of the PLA Joint Logistics Support Force, Ningbo, Zhejiang, China; 3 School of Medicine, Shaoxing University, Shaoxing, Zhejiang, China; 4 Ningbo College of Health Sciences, Ningbo, Zhejiang, China

**Keywords:** angiogenesis, burn wound healing, CTLA4Ig, gene therapy, immunomodulation, inflammation, platelet-rich fibrin, synergistic effect

## Abstract

**Background:**

Deep second-degree burns impair skin regeneration and carry high risks of scarring and infection. Achieving healing with minimal immune rejection remains challenging. Platelet-rich fibrin (PRF), an autologous biomaterial, promotes angiogenesis and repair via sustained growth factor release. CTLA4Ig, an immunomodulatory agent, can suppress T-cell-mediated rejection. We hypothesized that combining PRF with CTLA4Ig gene-transfected porcine skin would synergistically enhance wound healing by concurrently stimulating regeneration and modulating local immunity.

**Methods:**

A standardized deep second-degree burn was created on the dorsum of 32 Sprague-Dawley rats, randomly divided into four groups (n=8): Vaseline group, PRF group, Pigskin group, and PRF+pigskin group. Wound closure was tracked macroscopically for 21 days. Histological analysis (H&E, Masson’s trichrome), immunohistochemistry for CD31, VEGF, the pro-inflammatory cytokines IL-6 and TNF-α, and immunofluorescence staining for the antioxidant enzymes catalase (CAT) and superoxide dismutase 1 (SOD1) were performed on days 4, 7, 14, and 21.

**Results:**

The combination treatment (PRF+pigskin group) demonstrated a significant acceleration in wound closure compared to all other groups, with near-complete re-epithelialization observed by day 14. Statistical analysis confirmed a significant interaction between Treatment and Time (p<0.001), suggesting a synergistic healing pattern. Histological examination revealed more organized and dense collagen fibers, with the most pronounced effect in PRF+pigskin group. Immunohistochemical and immunofluorescence analyses indicated a marked upregulation of CD31-positive vessels, VEGF expression, and antioxidant enzymes (CAT and SOD1) in the combination group, indicating a trend towards enhanced angiogenesis and an augmented capacity to mitigate oxidative stress. Concurrently, immunohistochemistry for IL-6 and TNF-α revealed a significant attenuation of these pro-inflammatory cytokines in the PRF+pigskin group and the pigskin group, particularly at the later stages of healing (D14, D21), indicating a modulation of the local inflammatory response.

**Conclusion:**

The concomitant application of PRF and CTLA4Ig gene-transfected porcine skin suggests a synergistic effect, creating a pro-regenerative, immunomodulatory, anti-inflammatory, and antioxidative microenvironment. This resulted in significantly accelerated and improved healing of deep second-degree burn wounds, representing a promising and innovative therapeutic paradigm for the management of severe burns.

## 1 Introduction

Burn injuries represent a major global health burden, with deep second-degree burns constituting a particularly complex clinical entity (1). These burns are characterized by the destruction of the entire epidermis and a significant portion of the dermis, including hair follicles, sweat glands, and other dermal appendages. The inherent healing process for such injuries is often protracted, fraught with complications such as infection, excessive inflammation, aberrant collagen deposition leading to hypertrophic scarring, and functional impairment (2). The ultimate goal of burn wound management is to achieve rapid, cosmetically acceptable, and functional skin regeneration while minimizing contracture and scar formation (3).

The standard of care for deep partial-thickness burns has evolved beyond simple coverage with passive dressings like Vaseline gauze (4). The paradigm has shifted towards the use of bioactive wound coverings (5) that actively participate in the healing process. Biological dressings, particularly xenografts like porcine skin, have been used for decades as temporary wound covers. They provide a barrier against infection, reduce pain and fluid loss, and prepare the wound bed for eventual autografting. However, their widespread application is limited by the inevitable host immune rejection response (6), which can exacerbate local inflammation and impede the healing process.

To address the issue of immunogenicity, genetic engineering of donor tissues offers a compelling solution. Cytotoxic T-lymphocyte-associated protein 4 (CTLA4) is a pivotal immune checkpoint molecule that functions as a negative regulator of T-cell activation (7). CTLA4Ig, a recombinant fusion protein, binds to CD80/CD86 on antigen-presenting cells with high affinity (8), effectively blocking the CD28-mediated co-stimulatory signal necessary for full T-cell activation. The use of CTLA4Ig, including its gene therapy delivery, has been explored in transplantation and autoimmune contexts to induce immune tolerance (9, 10). The local expression of CTLA4Ig via gene-transfected porcine skin grafts represents a sophisticated strategy to create an immunoprivileged microenvironment at the wound site, thereby dampening the adaptive immune response against the xenogeneic tissue and allowing it to function longer as a bioactive scaffold. Furthermore, excessive inflammation and the associated oxidative stress are hallmarks of burn wounds that impair healing by damaging cells and tissues (11, 12). Therefore, evaluating the antioxidant capacity of a combined therapy provides crucial mechanistic insight into its overall efficacy. Strategies that combine immunomodulation with antioxidative and pro-regenerative stimuli are therefore highly desirable.

Concurrently, the field of regenerative medicine has embraced the use of autologous platelet concentrates for their potent wound-healing properties. Platelet-rich fibrin (PRF) is a second-generation platelet concentrate that has gained considerable attention. It is a completely autologous, leukocyte-rich fibrin matrix created through the slow polymerization of fibrinogen without the addition of bovine thrombin or other exogenous agents (13). This natural polymerization process results in a three-dimensional architecture that efficiently entraps platelets, leukocytes, and a myriad of growth factors, including platelet-derived growth factor (PDGF), transforming growth factor-β (TGF-β), vascular endothelial growth factor (VEGF), and epidermal growth factor (EGF) (9, 10). The gradual and sustained release of these bioactive molecules over 7–14 days makes PRF an ideal biomaterial for promoting key phases of wound healing (14): it enhances angiogenesis, stimulates fibroblast proliferation and migration, facilitates extracellular matrix (ECM) deposition (15, 16), and exhibits antimicrobial properties due to the presence of leukocytes.

While both CTLA4Ig-transfected xenografts and PRF have individually demonstrated efficacy in improving wound healing outcomes, their combination presents a novel, synergistic therapeutic approach. We hypothesize that CTLA4Ig-transfected porcine skin will provide a temporary, immunologically tolerated biological dressing that actively modulates the wound environment. Simultaneously, the application of PRF underneath the xenograft will directly supply a high concentration of growth factors and cytokines to the wound bed, powerfully stimulating angiogenesis, granulation tissue formation, and re-epithelialization from the wound margins and any remaining dermal appendages. The PRF membrane may also serve as an ideal interface between the host wound bed and the xenogeneic graft, further enhancing graft take and integration. Therefore, this study was designed to systematically evaluate the individual and combined effects of PRF and CTLA4Ig gene-transfected porcine skin on the healing of deep second-degree burns in a rat model. We assessed the outcomes through macroscopic wound closure rates, histological analysis of tissue architecture and collagen deposition, and immunohistochemical evaluation of angiogenic markers. Our findings aim to establish a proof-of-concept for this combinatory therapy as a superior strategy for managing deep burn wounds.

## 2 Materials and methods

A total of 32 male Sprague-Dawley (SD) rats, aged 6–8 weeks and weighing 250–300 g, were used in this study. All animals were housed under standard laboratory conditions (12-h light/dark cycle, 22 ± 2 °C, 50 ± 10% humidity) with ad libitum access to food and water. The study protocol was approved by the Institutional Animal Care and Use Committee of Zhejiang Huitong Test & Evaluation Technology Group Co., Ltd ([Approval No: HT-2025-LWFB-0036]), and all procedures were conducted in strict accordance with the National Institutes of Health Guide for the Care and Use of Laboratory Animals (17). The animal use permit number is SYXK (Zhe) 2022-0041.

### 2.2 Preparation and characterization of PRF

#### 2.2.1 Blood donor animals

Forty-eight healthy, specific pathogen-free (SPF) Sprague-Dawley rats (weight 250–300 g) served as blood donors for PRF preparation (18). These rats were housed under standard laboratory conditions for one week prior to the procedure to ensure acclimatization and health status. Rats exhibiting any signs of illness or stress were excluded.

#### 2.2.2 Rat anesthesia

Rats were anesthetized by intraperitoneal injection of Avertin (tribromoethanol, 2% solution, 1.5 ml/100 g body weight). A 2% Avertin anesthetic solution (1.5 ml/100 g body weight) was prepared in advance: 1g of tribromoethanol powder was weighed, 1ml of tert-amyl alcohol was added, and stirred thoroughly until completely dissolved. The solution was filtered through a 0.22μm filter membrane for sterilization, aliquoted into sterile reagent bottles, and stored at 4 °C, returning to room temperature before use. The anesthetic working solution was prepared at a ratio of Avertin stock solution: normal saline = 1:40.

Before anesthesia, rats were fasted for 6 hours (to prevent vomiting and aspiration after anesthesia) but not water-restricted. Rats were placed in a transparent housing box, restrained, and injected with the 2% 1.5 ml/100 g dose of Avertin anesthetic.

After injection, rats were placed in an observation cage for 5–10 minutes to assess anesthetic effect: disappearance of corneal reflex, relaxation of limb muscles, and no response to hind limb pinprick were considered satisfactory anesthesia. If the anesthetic effect was insufficient (e.g., rats still had limb movement, corneal reflex present), an additional 1/3 dose of 2% Avertin anesthetic was administered, avoiding excessive dosage leading to overdose.

#### 2.2.3 Abdominal aorta blood collection

Blood collection instruments were prepared: sterile hemostatic forceps, tweezers, 10ml sterile syringe, 20G sterile needle, sterile gauze.

The satisfactorily anesthetized rat was fixed in a supine position on the operating table. The abdominal skin was thoroughly disinfected with 75% alcohol cotton balls, wiping the area from the xiphoid process to the pubic symphysis three times.

Using sterile tweezers, the rat’s abdominal skin was lifted, and a 1-2cm longitudinal incision was made along the midline of the abdomen. Subcutaneous tissue and muscle layers were sequentially separated to expose the abdominal aorta (located anterior to the spine, presenting as a pulsating cord-like structure).

The abdominal aorta was gently lifted with hemostatic forceps. The syringe needle was inserted into the abdominal aorta at a 30° angle, and blood was drawn, collecting 8-10ml per rat (avoiding rapid blood collection causing vascular collapse, blood collection time controlled within 1 minute). If blood showed a tendency to clot during collection, the syringe angle was adjusted appropriately to ensure the needle remained within the vessel.

#### 2.2.4 PRF acquisition and processing

Centrifugation equipment was prepared: a desktop centrifuge, calibrated in advance (ensuring accurate centrifugal force of 408×*g* [relative centrifugal force, RCF]), centrifugation time set to 14 minutes, centrifuge temperature pre-adjusted to room temperature (to avoid temperature effects on PRF components).

The collected rat abdominal aortic blood was immediately transferred into sterile centrifuge tubes, 5ml blood per tube (avoiding excessive or insufficient blood volume affecting the effect of centrifugation). Following abdominal aortic puncture, whole blood was collected directly into sterile, non-coated 5 ml centrifuge tubes. For each individual donor rat, the elapsed time from the completion of the blood draw to the moment the centrifuge rotor reached its set speed of 408×g was rigorously standardized and recorded. This critical interval was consistently maintained at under 120 seconds (≤2 minutes). This rapid processing protocol was implemented to minimize pre-centrifugation clot formation, thereby ensuring the reproducible generation of a PRF clot characterized by a dense, three-dimensional fibrin architecture optimally entrapping platelets, leukocytes, and the associated cocktail of growth factors and cytokines. Tube caps were tightened, tube weights were balanced (error ≤ 0.1g), and tubes were placed symmetrically in the centrifuge rotor.

The centrifuge was started and operated at 408×*g* for 14 minutes. The process was uninterrupted, and parameters were not adjusted. After centrifugation, the centrifuge was turned off. After the rotor came to a complete stop, centrifuge tubes were slowly removed. Three distinct layers were visible: upper light yellow serum layer, middle milky white PRF gel layer, and lower deep red blood cell gel layer.

The upper serum layer was gently removed with sterile tweezers (serum could be collected for subsequent related tests). The lower red blood cell gel layer was then cut away along the tube wall using sterile scissors, avoiding damage to the middle PRF gel layer.

The middle PRF gel was grasped with sterile tweezers and placed in a sterile Petri dish. The PRF gel was wrapped in sterile gauze and pressed with moderate force (force not damaging the PRF gel structure) to absorb excess serum until no significant liquid seeped from the PRF gel surface. The PRF gel was then shaped into a membrane disc of 2cm diameter and 2mm thickness, placed in a sterile Petri dish, and stored at 4 °C for use within 24 hours.

#### 2.2.5 PRF morphological characterization

**Figure 1:**
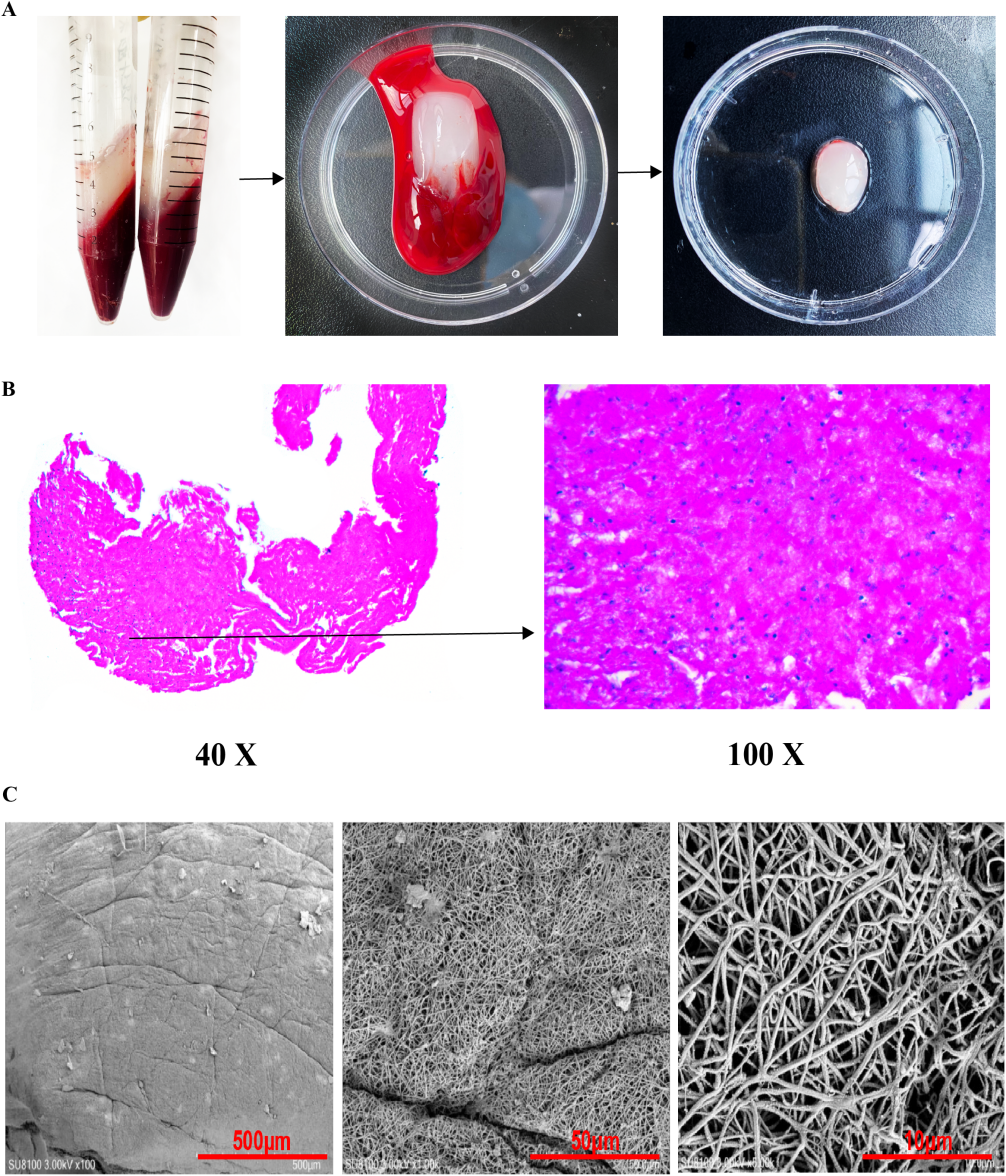
Preparation and characterization of autologous platelet-rich fibrin. **(A)** Schematic diagram and macroscopic view illustrating PRF membrane preparation; **(B)** Hematoxylin and Eosin (H&E) staining of PRF at (i) 40× and (ii) 100× magnification. The images display a dense, three-dimensional fibrin network (asterisks) with platelets and leukocytes embedded within the meshwork (characterized by round/oval nuclei scattered in the fibrin matrix). Scale bars: 500 μm (i), 50 μm (ii); **(C)** Scanning Electron Microscopy (SEM) image (3000× magnification) detailing the porous microarchitecture of the fibrin matrix, which is critical for sustained growth factor release.

The prepared autologous PRF membranes were characterized to confirm their structural integrity and typical composition, which are essential for their function as a growth factor delivery scaffold. Macroscopically, the compressed membranes appeared as resilient, yellowish, elastic fibrin clots (**Figure 1A**).

Histological examination using Hematoxylin and Eosin (H&E) staining confirmed the characteristic microstructure of PRF. Under light microscopy, the PRF membrane exhibited a dense, three-dimensional fibrin network serving as a scaffold. Within this network, numerous platelets and leukocytes were visibly entrapped, consistent with the expected composition of leukocyte-rich PRF (**Figure 1B**).

The ultrastructure of the fibrin matrix was further analyzed by Scanning Electron Microscopy (SEM). SEM images revealed an intricate, porous architecture formed by thin fibrin fibers. This specific microstructure is crucial as it provides the surface area and matrix necessary for the gradual entrapment and sustained release of platelets, leukocytes, and their contained bioactive factors over time (**Figure 1C**).

The detailed, step-by-step protocols for H&E staining and SEM sample processing are provided in Supplementary File S1.

### 2.3 Establishment of SD rat dorsal skin deep second-degree burn model and grouping

#### 2.3.1 Experimental animals and ethical approval

Thirty-two 6–8 week-old male Sprague-Dawley (SD) rats (weighing 250–300 g) were purchased from Beijing Sibefu Experimental Animal Co., Ltd. (Production License: SCXK (Jing) 2024-0001). Male rats were selected to minimize potential confounding effects of the female estrous cycle on skin physiology and wound healing (19). All animals were housed under standard laboratory conditions (12-h light/dark cycle, 22 ± 2 °C, 50 ± 10% humidity) with ad libitum access to food and water. After a 3-day acclimatization period, with daily health monitoring to screen for any pre-existing skin conditions or other abnormalities, the rats were randomly numbered (1-32) by ear tagging one day prior to the experiment for subsequent grouping. All procedures were approved by the Institutional Animal Care and Use Committee of Zhejiang Huitong Test & Evaluation Technology Group Co., Ltd ([Approval No: HT-2025-LWFB-0036]) and conducted in accordance with the institutional guidelines for animal welfare ([Animal Use License: SYXK (Zhe) 2022-0041]).

#### 2.3.2 Rat dorsal hair removal

Before depilation, rats were placed in restrainers, heads and limbs gently fixed to avoid struggle. Dorsal hair was cut short to 1-2mm with sterile scissors (avoiding long hair affecting depilatory cream effect), careful to avoid scissor cuts.

A depilatory cream (Veet) was applied evenly with a sterile cotton swab to the dorsal depilation area (approx. 5cm×5cm, ensuring coverage of subsequent burn creation area), thickness sufficient to completely cover hair, avoiding flow to other areas (eyes, abdominal skin).

After application, left for 3–5 minutes, observing hair dissolution: when hair roots turned white and could be wiped off gently, the dorsal skin was immediately rinsed thoroughly with sterile distilled water for 5–10 minutes, ensuring complete removal of residual sodium sulfide solution to avoid skin burns.

After rinsing, dorsal skin was gently dried with sterile gauze, observed for redness, swelling, or damage. If mild redness occurred, a small amount of Vaseline was applied, waiting for skin to return to normal before proceeding; if skin damage occurred, the rat was excluded and replaced with a spare.

#### 2.3.3 Deep second-degree burn wound creation

##### 2.3.3.1 Pre-creation preparation

Burn instruments: flat-bottom glass tube (a custom-made brass rod with a 20-mm diameter contact surface) diameter 20mm, height 100mm (pre-washed with distilled water, dried), constant temperature water bath, sterile normal saline, 75% alcohol cotton balls, sterile gauze, marker pen, ruler.

Water bath temperature set to 95 °C, sufficient distilled water added, waiting for temperature stabilization at 90-100 °C (monitored with thermometer).

Satisfactorily anesthetized rat fixed in prone position on experimental table, sterile gauze padded under chest and abdomen to fully extend and flatten dorsal skin, avoiding folds affecting wound size/depth.

Dorsal depilated area disinfected with 75% alcohol cotton balls, wiping from center outwards 3 times. After alcohol evaporation, burn center point measured and marked with ruler (on dorsal midline, approx. 2cm from scapulae).

##### 2.3.3.2 Burn wound creation

A flat-bottom glass tube (diameter 2cm) was grasped with tweezers, mouth down, vertically placed into the constant temperature water bath, filled with 90-100 °C hot water, held for 5 minutes ensuring tube wall temperature reached 90-100 °C.

Tube removed, external water quickly wiped dry. The tube mouth was pressed firmly against the marked site for 40 seconds to ensure complete and gap-free skin contact.

After 40s, tube quickly removed. A circular pale area appeared on dorsal skin, the burn wound. A 2cm diameter circular area accurately marked along wound edge with marker pen (aided by ruler).

After creation, wound and surrounding skin gently wiped with sterile normal saline to remove potential residue. Wound condition observed: normal deep second-degree burn wound appears pale or light brown, dry, no or few small blisters, slight pain on touch (partial nerve ending damage). If extensive blisters, charring (possibly third-degree), or only mild redness (possibly superficial second-degree), model failed, rat excluded, model recreated.

##### 2.3.3.3 Pathological section verification of wound depth

To confirm the successful establishment of the deep second-degree burn model, wound tissue samples (including the wound edge and adjacent normal skin) were collected from three randomly selected rats 24 hours post-burn. The samples were fixed in 4% paraformaldehyde, processed, and embedded in paraffin. Sections (4 μm) were stained with H&E and examined under a light microscope. Histological characteristics of a deep second-degree burn, including full-thickness epidermal necrosis, necrosis of the superficial to mid-dermis, the presence of residual dermal appendages (e.g., hair follicles, sweat glands), and inflammatory cell infiltration in the dermis, were observed in all samples, confirming the consistent and successful creation of the intended burn wound depth.

#### 2.3.4 Grouping and treatment methods

##### 2.3.4.1 Random grouping

Thirty-two rats were randomly divided into 4 groups, with 8 rats in each group: Vaseline group (Control), PRF group (PRF + Vaseline group), Pigskin group (CTLA4Ig gene-transfected porcine skin group), and PRF+pigskin group (PRF + CTLA4Ig gene-transfected porcine skin group). The grouping was random and balanced. For terminal histological and molecular analyses, a subset of 2 rats per group was allocated at each time point.

##### 2.3.4.2 Wound treatment for each group

###### 2.3.4.2.1 General preoperative preparation

The required dressings and instruments for each group were prepared: Vaseline medical gauze, PRF membranes (freshly prepared as described previously, soaked in sterile normal saline for 10 seconds before use for moisture), CTLA4Ig gene-transfected porcine skin (manufacturer: Chongqing Keji Biotechnology Co., Ltd.; Approval Number: National Medical Device Approval 20143142325) was used. This product is a biologically active dressing constructed from Bama mini-pig skin tissue genetically transfected with the human CTLA4Ig gene. The dressing was aseptically processed using a composite sterilization method (including ethanol, iodophor, UV irradiation, and benzalkonium bromide immersion) and supplied cryopreserved in -20 °C refrigerator with a shelf life of 2 years. For use, the graft was thawed in 37 °C sterile normal saline approximately 2 hours before application, with gentle agitation during thawing, sterile gauze, elastic net sleeves, iodophor solution (0.5%), sterile normal saline, sterile scissors, tweezers, and 1ml syringes.

Rats were fixed in sequence according to their numbers, and the anesthetic effect was rechecked. The wound and surrounding 2 cm of skin were disinfected with iodophor solution, wiping spirally from the center outwards 3 times. After the iodophor dried, the wound was gently rinsed with sterile normal saline to remove residue, and the surface moisture was blotted dry with sterile gauze.

###### 2.3.4.2.2 Vaseline group treatment

Three layers of sterile gauze were placed on the Vaseline surface, ensuring complete coverage of the wound and surrounding 1 cm of skin, and gently pressed with sterile tweezers for close contact. Two sheets of sterilized Vaseline gauze were applied, followed by 3 layers of sterile gauze.

An appropriately sized elastic net sleeve was selected, placed over the rat covering the dorsal wound area, and the position was gently adjusted to prevent gauze displacement.

###### 2.3.4.2.3 PRF group treatment

After completing the general preparation steps, a prepared PRF membrane (trimmed into a 2.5 cm diameter circle, slightly larger than the wound) was grasped with sterile tweezers. The rough side (the side facing the red blood cells) was placed facing down onto the wound, and the edges were gently pressed to ensure tight contact without air bubbles.

Following the method for Vaseline group, Vaseline gauze and sterile gauze were applied over the PRF membrane and secured with an elastic net sleeve. Vaseline was used in Vaseline group and PRF group as a standard, inert wound contact layer and occlusive covering to maintain a moist environment, a common practice in rodent burn studies (20). Its use in PRF group allowed for the isolation of PRF’s effects when compared to the inert control (Vaseline group) and the active biological dressing groups (Pigskin group and PRF+pigskin group).

###### 2.3.4.2.4 Pigskin group treatment

Thawed CTLA4Ig gene-transfected porcine skin: After thawing to 37 °C, the porcine skin was removed. Its surface was gently rinsed with sterile normal saline to remove any potential residual preservative solution, and the surface moisture was blotted dry with sterile gauze.

####### 2.3.4.2.4.1 Porcine skin perforation

Using sterile scissors, drainage holes were punched at intervals of 0.5 cm across the entire graft. The diameter of each hole was approximately 2 mm. This perforation procedure was performed to ensure adequate drainage of wound exudate, thereby preventing fluid accumulation or pus formation beneath the graft, while taking care not to damage the dermal structure.

####### 2.3.4.2.4.2 Graft application

The perforated porcine skin was trimmed into a circle of 3 cm in diameter. It was then applied to the wound with the dermal side facing down, ensuring complete coverage of the wound and the surrounding 0.5 cm of skin. The edges were gently pressed to achieve tight contact with the wound bed. Finally, the porcine skin was sutured to the adjacent normal skin at its four edges for secure fixation.

Following the method for Vaseline group, Vaseline gauze and sterile gauze were applied over the porcine skin and secured with an elastic net sleeve.

###### 2.3.4.2.5 PRF+pigskin group treatment

After completing the general preparation steps, the PRF membrane was first applied to the wound as described for PRF group.

Then, following the method described for Pigskin group, the thawed, perforated, and trimmed CTLA4Ig gene-transfected porcine skin graft was applied over the PRF membrane, ensuring tight contact between the two layers and with the wound bed.

Following the method for Vaseline group, Vaseline gauze and sterile gauze were applied over the porcine skin and secured with an elastic net sleeve.

##### 2.3.4.3 Postoperative observation

Wound dressings were changed every 48 hours under light anesthesia. During each dressing change, the wound was gently cleansed with sterile normal saline to remove exudate and debris before the application of new treatments. The rats’ mental state, appetite, water intake, activity levels, and the skin around the wound for redness or signs of inflammation spread were observed daily. Weight changes were recorded weekly. If death occurred, a necropsy was performed promptly to analyze the cause, and rats from the same batch were supplemented to maintain the required number.

##### 2.3.4.4 Specimen collection

At predetermined time points (post-burn days 4, 7, 14, and 21), two rats per group were randomly selected and euthanized for tissue sampling. Euthanasia was performed by intraperitoneal injection of a 3-fold routine dose of Avertin (21). Following euthanasia, the dorsal skin was thoroughly disinfected with 75% alcohol. Full-thickness skin samples encompassing the wound center and adjacent edge (1 cm × 1 cm × 0.3 cm, including 0.5 cm of surrounding normal tissue) were aseptically excised. Each sample was divided into two portions: one was fixed in 4% paraformaldehyde for subsequent histological processing (Masson’s trichrome staining and immunohistochemistry), and the other was snap-frozen in RNA later at –80 °C for potential molecular analyses. All specimens were clearly labeled with group, animal identifier, and collection date.

A note on sample size for quantitative histology: The sample size for histological, immunohistochemical, and immunofluorescence quantitative assessments was n = 2 biological replicates (rats) per group per time point. To enhance the robustness of the data, multiple sections were analyzed per sample, and several non-overlapping fields were evaluated per section. The mean value derived from all measurements for each rat was used as the statistical unit for inter-group comparisons.

### 2.4 Wound healing assessment

#### 2.4.1 Macroscopic evaluation

The wounds were photographed on days 0, 2, 4, 7, 14, and 21 using a digital camera with a fixed scale. The wound area was measured using ImagePro Plus 6.0 software. The wound healing rate was calculated as follows: Wound Healing Rate (%) = [(Initial Wound Area - Wound Area at Time Point)/Initial Wound Area] × 100%.

### 2.5 Histological and immunohistochemical analysis

The harvested wound tissues were fixed in 4% paraformaldehyde, embedded in paraffin, and sectioned into 5 μm thick slices.

#### 2.5.1 H&E and Masson’s trichrome staining

H&E staining was performed to evaluate general histological morphology, including the degree of re-epithelialization, granulation tissue thickness, and inflammatory cell infiltration. Masson’s trichrome staining was used specifically to assess collagen deposition, organization, and maturation within the dermis. The detailed staining protocol is provided in Supplementary File S1. Stained sections were examined under a light microscope (Nikon Eclipse E100), and representative images were captured.

#### 2.5.2 Quantitative histomorphometry

##### 2.5.2.1 Collagen volume fraction

The CVF was quantified from Masson’s trichrome-stained sections using ImageJ software. For each section, five random, non-overlapping fields of view (200x magnification) from the central dermal granulation tissue area were selected. The percentage of the blue-stained collagen area relative to the total tissue area in each field was calculated, and the average CVF per sample was determined.

##### 2.5.2.2 Re-epithelialization

The percentage of re-epithelialization was measured from H&E-stained sections by calculating the length of the newly formed epidermis relative to the total wound gap length.

#### 2.5.3 Immunohistochemistry for CD31, VEGF, IL-6, and TNF-α

IHC staining for all protein markers (CD31, VEGF, IL-6, TNF-α) was performed following a standard Streptavidin-Peroxidase (SP) method. Briefly, after deparaffinization, rehydration, and antigen retrieval (citrate buffer, pH 6.0, for CD31 and VEGF; EDTA buffer, pH 9.0, for IL-6 and TNF-α), sections were incubated overnight at 4 °C with the respective primary antibodies: Rabbit anti-rat CD31 (1:500, Cat# 33075-1-AP, Proteintech), Rabbit anti-rat VEGF (1:500, Cat# 26157-1-AP, Proteintech), Rabbit anti-rat IL-6 (1:500, Cat# 21865-1-AP, Proteintech), and Rabbit anti-rat TNF-α (1:500, Cat# 17590-1-AP, Proteintech). Negative controls were processed by omitting the primary antibody. Following incubation with a biotinylated Goat Anti-Rabbit IgG secondary antibody (1:200, Cat# HA1001, Hua’an Biotechnology) and a Streptavidin-HRP complex, the signal was visualized using a DAB substrate kit. Sections were counterstained with hematoxylin.

#### 2.5.4 Immunofluorescence staining for antioxidant enzymes (CAT and SOD1)

The protocol for IF staining is described in detail in Section 2.8.

#### 2.5.5 Quantitative IHC/IF analysis

All quantitative analyses were performed by two independent investigators who were blinded to the group assignments.

For CD31, microvessels were identified as any brown-stained endothelial cell or cluster separate from adjacent vessels. Microvessel density (MVD) was expressed as the average count of CD31-positive structures per high-power field (HPF, 200x) from five random HPFs within the granulation tissue per section.

For VEGF, IL-6, and TNF-α, expression levels were quantified using ImageJ software. For VEGF, the percentage of DAB-positive (brown) area relative to the total tissue area was measured in five random HPFs (200x). For IL-6 and TNF-α, the average optical density (AOD) of the DAB-positive area was measured in five random HPFs (200x).

For IF analysis of CAT and SOD1, the average fluorescence intensity was measured in five random fields (200x) within the granulation tissue using ImageJ.

To facilitate comparison across different time points and groups, the raw quantitative values (MVD, VEGF Positive Area %, IL-6 AOD, TNF-α AOD, Fluorescence Intensity) for each sample were normalized to the mean value of the Vaseline group at Day 4, which was set to 1.00.

Given the exploratory nature and the sample size for these molecular analyses (n=2 biological replicates per group per time point), which limits statistical power, the quantitative data are presented as mean ± standard deviation (SD) to illustrate consistent trends. Statistical comparisons between groups for these parameters are interpreted with extreme caution and are not the primary basis for our conclusions.

### 2.6 Statistical analysis

The primary outcome, wound healing rate (%), measured longitudinally, was analyzed using a Linear Mixed-Effects Model (LMM) to appropriately account for the repeated-measures design and the correlation of data points within each subject (22). The model was fitted using the lmer function from the ‘lme4’ package (version 1.1-35.3) in R (version 4.3.0). The fixed effects structure included Treatment, Time (modeled as a categorical factor), and the Treatment × Time interaction. To account for the non-independence of repeated measurements, a random intercept for each Rat ID was included in the model, allowing each animal to have its own baseline healing rate. Model diagnostics (residual plots, Q-Q plots) confirmed no major violations of homoscedasticity or normality. Significance tests for fixed effects were performed using the Satterthwaite approximation for degrees of freedom via the lmerTest package. *Post-hoc* pairwise comparisons at individual time points, derived from the fitted model, were conducted using estimated marginal means with Tukey’s Honest Significant Difference (HSD) adjustment for multiple comparisons.

For the quantitative histological and IHC data (CVF, MVD, VEGF, IL-6, TNF-α, CAT, SOD1 intensity), given the smaller sample size (n=2) per group per time point, which limits statistical power, the data are primarily presented descriptively (mean ± SD) to illustrate consistent trends across the study timeline that support the macroscopic findings. A p-value of less than 0.05 was considered statistically significant.

### 2.7 Detailed experimental protocols

#### 2.7.1 PRF membrane preparation protocol

The PRF membrane was prepared following the standardized protocol described in sections 2.2.1-2.2.4, which includes anesthesia, abdominal aorta blood collection, centrifugation at 408×g for 14 minutes, clot extraction, and membrane formation. Detailed step-by-step procedures are provided in Supplementary File S1 (Section S1.1).

#### 2.7.2 Immunohistochemistry staining protocol

IHC staining was performed using the Streptavidin-Peroxidase (SP) method as previously described (9, 17)°Briefly, after deparaffinization, rehydration, and antigen retrieval, sections were incubated overnight at 4 °C with primary antibodies against CD31 (1:500, Proteintech), VEGF (1:500, Proteintech), IL-6 (1:500, Proteintech), and TNF-α (1:500, Proteintech). Following incubation with a biotinylated secondary antibody and a Streptavidin-HRP complex, the signal was visualized using DAB and counterstained with hematoxylin. Complete protocol details are available in Supplementary File S1 (Section S1.5).

### 2.8 Immunofluorescence staining for antioxidant enzymes

IF staining for CAT and SOD1 was conducted following standard procedures. After deparaffinization and antigen retrieval (similar to IHC), sections were permeabilized with 0.1% Triton X-100, blocked with 5% BSA, and incubated overnight at 4 °C with primary antibodies against CAT (1:200, Proteintech) and SOD1 (1:200, Proteintech). After washing, sections were incubated with a Cy3-conjugated secondary antibody (1:400) for 1 hour at room temperature. Nuclei were counterstained with DAPI. The complete protocol is detailed in Supplementary File S1 (Section S1.6).

## 3 Results

### 3.1 Characterization of PRF membranes

The autologous PRF membranes were successfully prepared from all experimental rats. Macroscopically, the membranes appeared as resilient, yellowish, and elastic fibrin clots following compression. Histological examination with H&E staining revealed the classic microstructure of PRF: a dense fibrin matrix serving as a scaffold that entrapped a significant number of platelets and leukocytes within its meshes. Scanning electron microscopy (SEM) at higher magnification further elucidated this intricate three-dimensional architecture, showcasing the fine fibrin network that is crucial for the gradual and sustained release of cytokines and growth factors over time (**Figure 1**).

### 3.2 Macroscopic evaluation of wound healing

**Figure 2:**
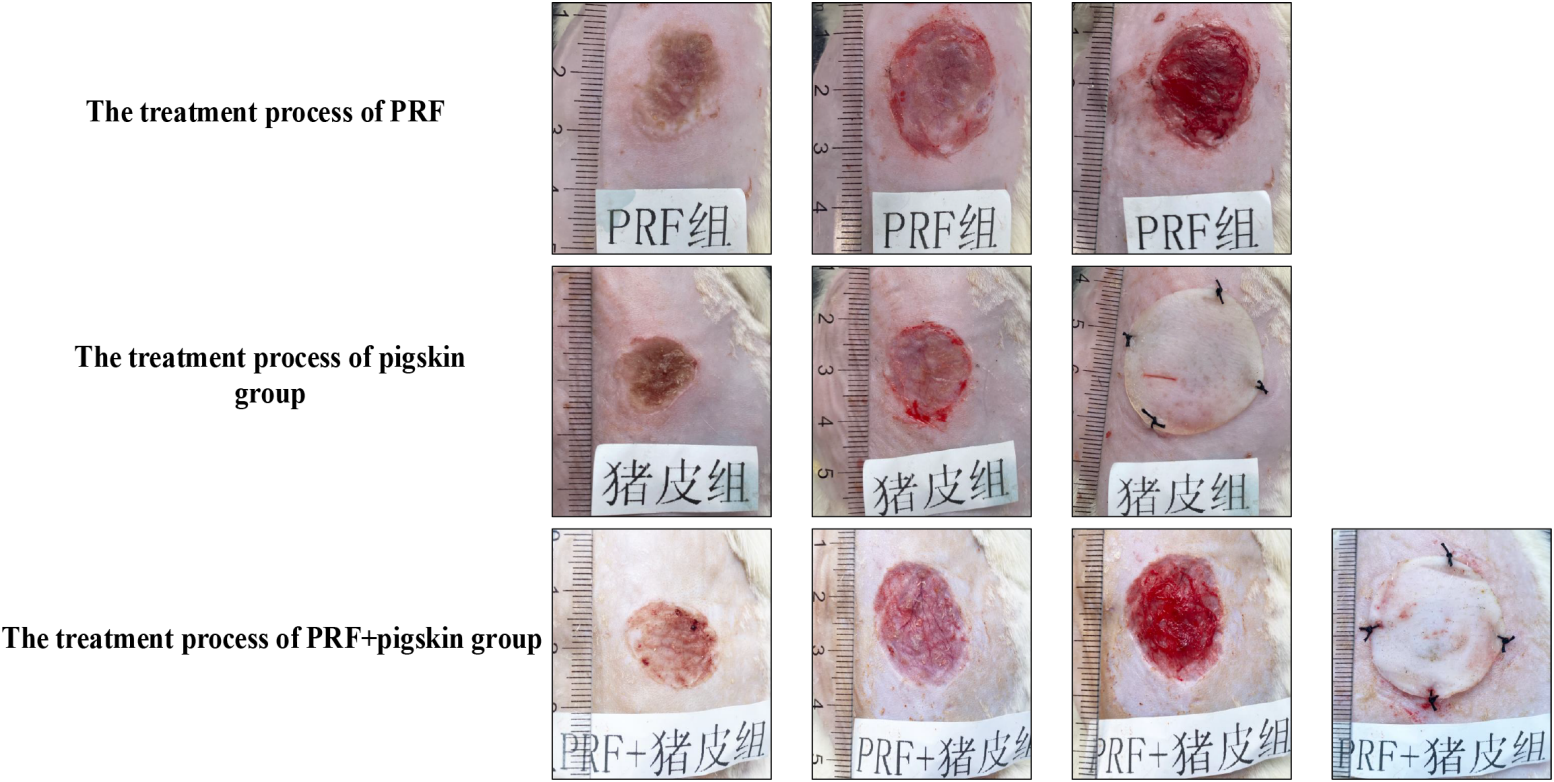
Procedural overview of wound treatments. Representative photographs illustrating the key steps of therapy application for each group. Top row (PRF group): (i) Burn wound after creation, (ii) Application of the PRF membrane to the wound bed, (iii) Wound after PRF application. Middle row (Pigskin group): (i) Burn wound after creation, (ii) Fitting of the perforated CTLA4Ig gene-transfected porcine skin graft, (iii) Wound after suturing the porcine skin graft in place. Bottom row (PRF+pig skin group): (i) Burn wound after creation, (ii) Application of the PRF membrane, (iii) Placement of the porcine skin graft over the PRF layer, (iv) Wound after securing the combined graft.

All animals tolerated the experimental procedures well and survived the entire study period without signs of severe systemic infection, as assessed by daily monitoring of activity, appetite, and the absence of purulent discharge or systemic illness. The dynamic process of wound contraction and re-epithelialization was documented macroscopically across all groups (**Figure 2**, **Supplementary Figure 1**).

#### 3.2.1 Vaseline group

Wounds in the control group exhibited the slowest healing trajectory. Prominent eschar formation and persistent inflammation were observed during the early phase (Days 4-7). Wound contraction was sluggish, and by Day 21, the wound areas remained significantly larger than those in all treatment groups, often with incomplete epithelialization.

#### 3.2.2 PRF group

Treatment with PRF alone significantly accelerated wound closure compared to Vaseline group. The PRF membrane integrated well with the wound bed, providing a moist environment that reduced surface exudation and facilitated the formation of cleaner, healthier granulation tissue. *Post-hoc* analysis following the LMM confirmed that the wound healing rate in PRF group was significantly higher than in Vaseline group from Day 7 onwards (p < 0.05).

#### 3.2.3 Pigskin group

The CTLA4Ig-transfected porcine skin graft provided effective biological coverage. It adhered satisfactorily to the wound bed with minimal signs of rejection or underlying complications such as seroma or abscess formation, supporting the local immunomodulatory role of CTLA4Ig. Wound contraction progressed steadily and *post-hoc* tests indicated it was significantly improved over Vaseline group by Day 14 (p < 0.05).

#### 3.2.4 PRF+pigskin group

The combination therapy yielded the most rapid and superior macroscopic healing outcome. Wounds in this group demonstrated minimal inflammation, rapid eschar dissolution, and the most accelerated rate of wound contraction. Near-complete re-epithelialization was typically achieved by Day 14, which was markedly earlier than in all other groups. The wound healing rate in PRF+pigskin group was statistically superior to that in Vaseline group at all post-operative time points (Days 4, 7, 14, and 21; p < 0.01). Furthermore, PRF+pigskin group demonstrated a significant enhancement in healing compared to both the PRF group and the pigskin group from Day 7 onwards (p < 0.05 for all comparisons), underscoring a differential therapeutic effect consistent with synergy.

### 3.3 Statistical modeling of synergistic effects

Statistical modeling of the longitudinal wound healing data using a Linear Mixed-Effects Model revealed significant main effects for Treatment (F(3, 28) = 40.15, p < 0.001) and Time (F(3, 84) = 105.32, p < 0.001). Most importantly, a highly significant Treatment × Time interaction was confirmed (F(9, 84) = 8.67, p < 0.001). This statistically sound interaction term formally validates that the effect of the therapeutic intervention on the rate of healing was not parallel but differed significantly across the post-burn timeline, which is the core statistical definition of an interaction effect supporting a differential healing trajectory. *Post-hoc* comparisons at each time point showed that the wound healing rate in PRF+pigskin group was statistically superior to that in Vaseline group at all post-operative time points (Days 4, 7, 14, and 21; p < 0.01). Furthermore, PRF+pigskin group demonstrated a significant enhancement in healing compared to both the PRF group and the Pigskin group from Day 7 onwards (p < 0.05 for all comparisons). Notably, the observed healing rate in PRF+pigskin group at Day 14 appeared to surpass what would be expected from a simple additive effect of the individual PRF group and Pigskin group treatments relative to Vaseline group, which is suggestive of a synergistic interaction (see **Table 1** for LMM results). The original two-way ANOVA results, which are provided in **Supplementary Table S2** for transparency, were consistent in showing significant main and interaction effects.

**Table 1:** Linear mixed-effects model results for the effects on wound healing rate.

Source	F-value (Num DF, Den DF)	P-value
Treatment (Fixed Effect)	F(3, 28) = 40.15	< 0.001
Time (Fixed Effect)	F(3, 84) = 105.32	< 0.001
Treatment × Time (Interaction)	F(9, 84) = 8.67	< 0.001

A linear mixed-effects model was fitted to the wound healing rate data, with Treatment (Vaseline group, PRF group, Pigskin group, PRF+pigskin group) and Time as fixed effects and a random intercept for each Rat ID. The significant interaction effect (p < 0.001) indicates that the effect of the treatment depended on the time point assessed. Data are derived from macroscopic wound area measurements (n=8 rats per group).

### 3.3 Histological analysis and collagen deposition

**Figure 3:**
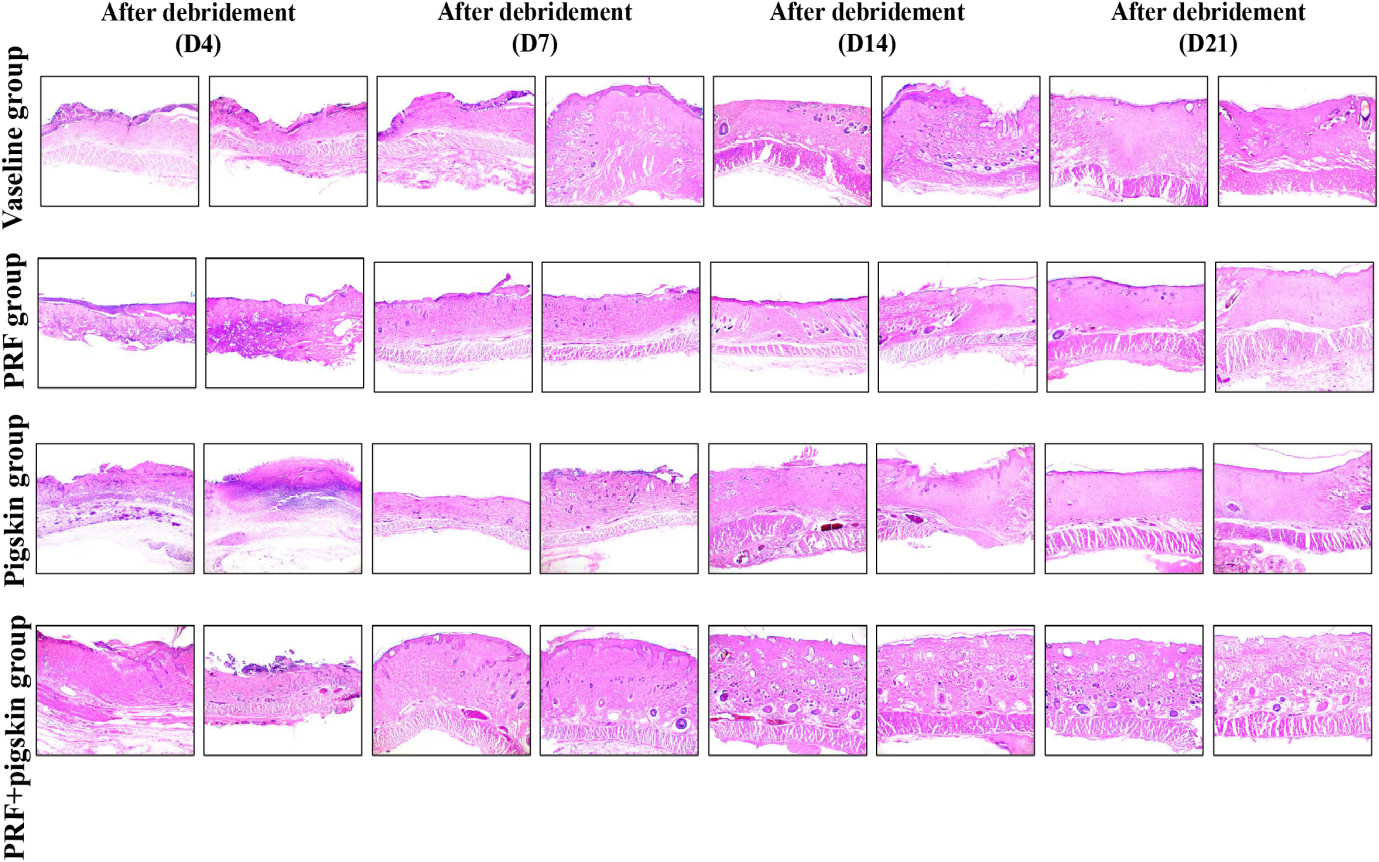
Histological evaluation of wound healing by Hematoxylin and Eosin (H&E) staining. Representative H&E-stained images of wound tissues from the Vaseline group, PRF group, Pigskin group, and PRF+pig skin group on Days 4, 7, 14, and 21 post-debridement. Inflammatory cell infiltrates are localized in the superficial layer of the granulation tissue. The leading edge of epithelial migration is defined as the advancing margin of the neo-epidermis extending toward the wound center. By Day 14, the PRF+pig skin group exhibits a thick, mature neo-epidermis, well-vascularized granulation tissue, and minimal inflammatory cell infiltration compared to the other groups, indicating advanced healing progression. Scale bar = 200 μm.

**Figure 4:**
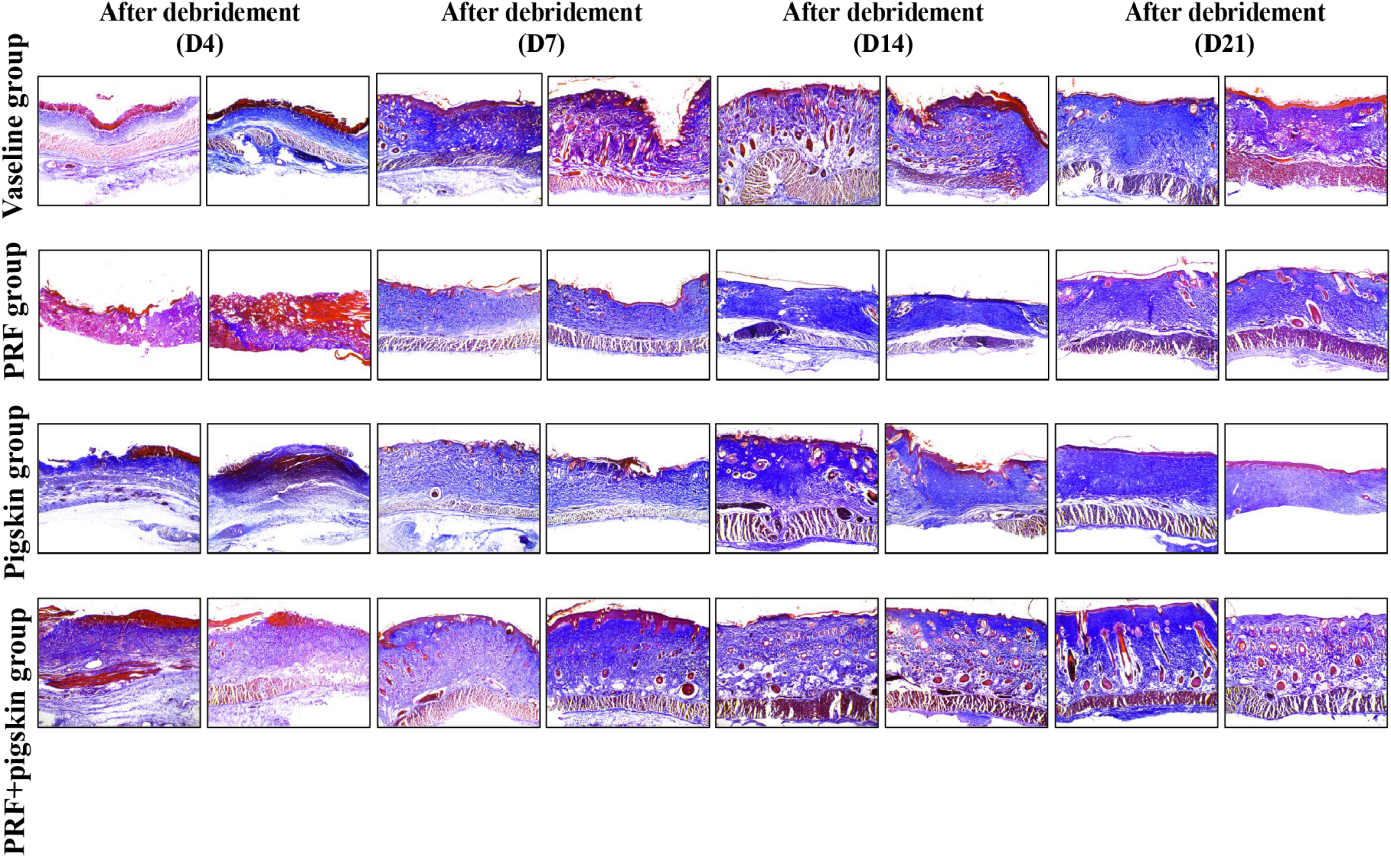
Collagen deposition and organization assessed by Masson’s trichrome staining. Collagen fibers are stained blue. Representative images from the Vaseline group, PRF group, Pigskin group, and PRF+pig skin group on Days 4, 7, 14, and 21 post-debridement. On Day 7, collagen in the Vaseline group is sparse and disorganized. In contrast, the PRF+pig skin group exhibits more abundant and better-organized collagen deposition as early as Day 7, progressing to dense, basket-weave-like collagen bundles by Days 14 and 21, indicating advanced tissue maturation. Scale bar = 100 μm.

Histological evaluation via H&E and Masson’s trichrome staining provided detailed insights into the healing quality (**Figures 3**, **4**).

H&E staining revealed that by Day 14, PRF+pigskin group exhibited the most advanced healing morphology, characterized by a thick, mature neo-epidermis, robust granulation tissue formation, and a notably reduced inflammatory cell infiltrate. In contrast, Vaseline group still displayed a thin, immature epithelium, persistent neutrophilic infiltration, and less developed granulation tissue.

Masson’s trichrome staining, which specifically highlights collagen deposits in blue, provided a clear assessment of dermal remodeling. On Day 7, collagen fibers in Vaseline group were sparse, fragmented, and randomly organized. At the same time point, PRF+pigskin group already demonstrated more abundant and better-organized collagen deposition. This difference became increasingly pronounced at later stages. By Days 14 and 21, the collagen fibers in PRF+pigskin group were densely packed, thick, and exhibited a basket-weave pattern reminiscent of normal dermis, indicating advanced maturation. The PRF group and Pigskin group showed intermediate levels of collagen organization and density.

Quantitative image analysis of the collagen volume fraction (CVF) corroborated these observations. The CVF data, presented in **Supplementary Table S1**, illustrate that the PRF+pigskin group consistently showed the highest values, particularly at Days 14 and 21, aligning with the qualitative maturation observed in Masson’s trichrome staining. Due to the sample size (n=2 per group per time point), these data are presented descriptively to illustrate trends consistent with the histological findings. The quantitative trend of CVF increase, illustrated in **Supplementary Figure 1**, was most pronounced in the PRF+pigskin group.

### 3.4 Immunohistochemical analysis of angiogenesis and VEGF expression

To elucidate the mechanisms behind the enhanced healing, we assessed angiogenesis (via CD31) and the expression of a key pro-angiogenic cytokine (VEGF). The quantitative trends are summarized in **Tables 2**, **3**.

**Table 2:** Quantitative analysis of normalized CD31 immunohistochemical staining (microvessel density or positive area %).

Group	Post-debridement D4	Post-debridement D7	Post-debridement D14	Post-debridement D21
Vaseline group	1.00 ± 0.00	0.58 ± 0.15	1.31 ± 0.10	0.90 ± 0.10
PRF group	3.01 ± 0.16	1.92 ± 0.11	2.13 ± 0.07	2.65 ± 0.10
Pigskin group	2.25 ± 0.14	2.83 ± 0.12	2.11 ± 0.07	1.76 ± 0.14
PRF+pigskin group	2.49 ± 0.09	2.85 ± 0.10	1.57 ± 0.20	1.88 ± 0.12

Data are presented as mean ± SD (n=2 biological replicates). Values are normalized to the Vaseline Group D4 mean. Due to the sample size, formal inferential statistics are not applied; data are shown to illustrate consistent trends across the study timeline.

**Table 3:** Quantitative analysis of normalized VEGF immunohistochemical staining (positive area %).

Group	Post-debridement D4	Post-debridement D7	Post-debridement D14	Post-debridement D21
Vaseline group	1.00 ± 0.00	1.48 ± 0.15	0.93 ± 0.07	1.29 ± 0.11
PRF group	3.12 ± 0.18	1.45 ± 0.07	2.16 ± 0.52	3.51 ± 0.14
Pigskin group	3.59 ± 0.33	1.84 ± 0.06	2.31 ± 0.12	2.56 ± 0.16
PRF+pigskin group	2.56 ± 0.15	2.45 ± 0.14	2.50 ± 0.09	3.08 ± 0.09

Data are presented as mean ± SD (n=2 biological replicates). Values are normalized to the Vaseline Group D4 mean. Due to the sample size, formal inferential statistics are not applied; data are shown to illustrate consistent trends across the study timeline.

**Figure 5:**
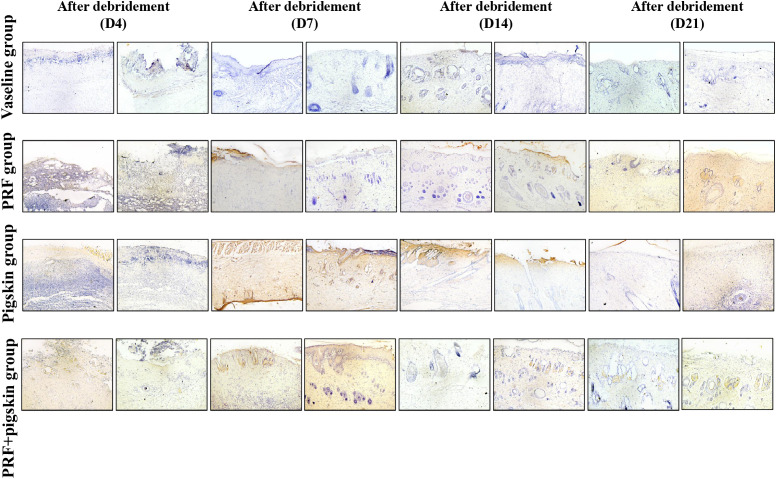
Immunohistochemical analysis of vascular endothelial growth factor expression. Representative images of VEGF staining (brown) in wound tissues from the Vaseline group, PRF group, Pigskin group, and PRF+pig skin group on Days 4, 7, 14, and 21. Robust cytoplasmic VEGF immunoreactivity, with a distribution pattern consistent with expression in fibroblasts, endothelial cells, and infiltrating mononuclear cells, is observed within the granulation tissue of the PRF group and PRF+pig skin group, particularly during the early and middle phases of healing. Scale bar = 100 μm.

Immunohistochemistry for CD31 revealed a greater number of brown-stained, CD31-positive microvessels within the granulation tissue of the PRF group and PRF+pigskin group on Days 7 and 14 compared to the Vaseline group and Pigskin group. The quantitative data (**Table 2**) showed that the PRF+pigskin group exhibited the highest normalized values at most time points, followed by the PRF group. The Pigskin group showed a moderate increase compared to the Vaseline group at later stages (D14, D21). Notably, the pronounced angiogenic effect was most consistent and sustained in the groups containing PRF (PRF group and PRF+pigskin group) (**Figure 5**).

**Figure 6:**
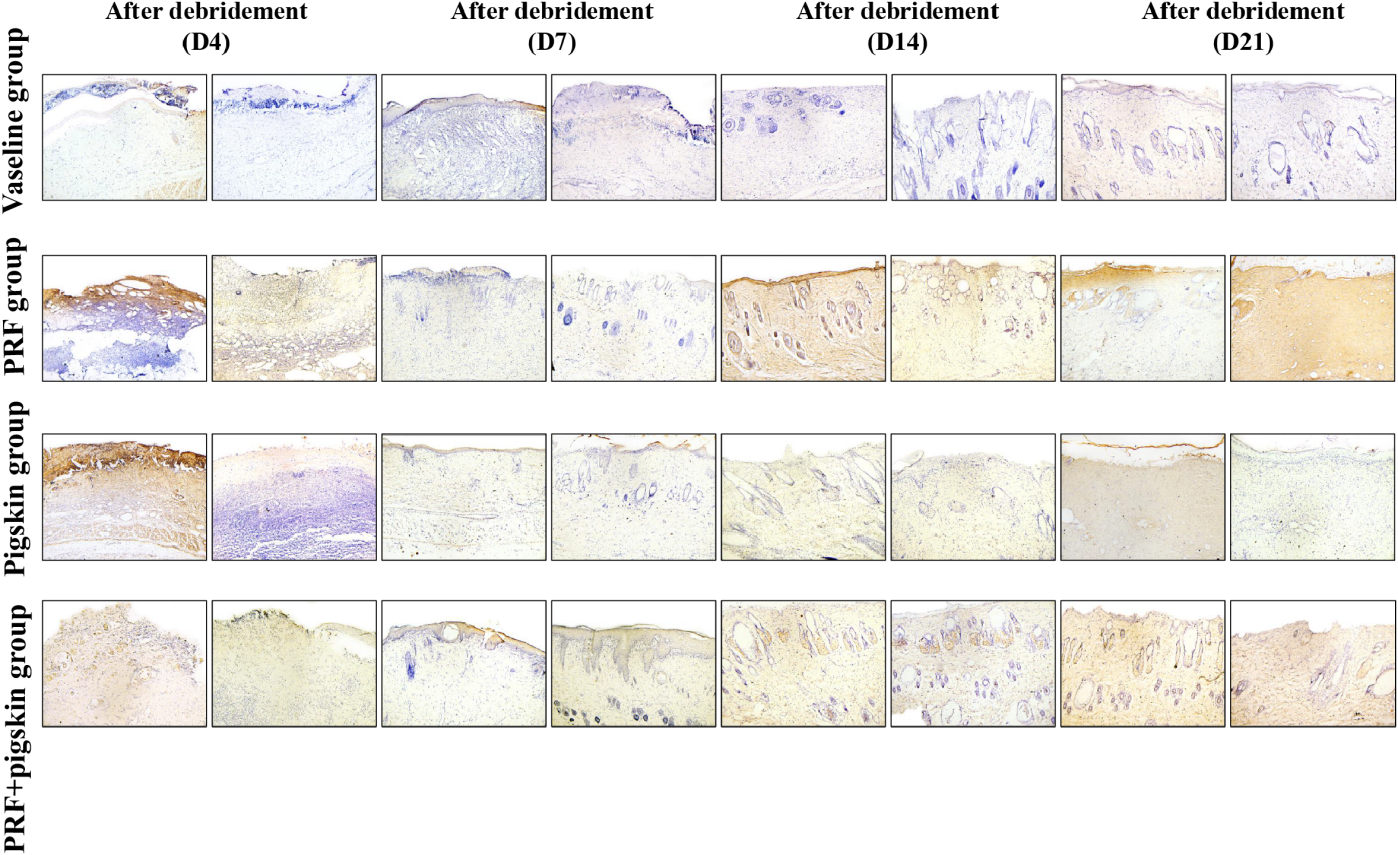
Immunohistochemical evaluation of angiogenesis via CD31 staining. Representative immunohistochemical images of CD31 staining (brown immunoreactivity, indicating vascular endothelial cells) in wound tissues from the Vaseline group, PRF group, Pigskin group, and PRF+pig skin group on Days 4, 7, 14, and 21 post-debridement. CD31-positive microvessels are identified as discrete brown-stained structures with lumen-like morphology (characterized by linear or circular arrangements of endothelial cells) distributed within the granulation tissue. The PRF group and PRF+pig skin group show a marked increase in the density of CD31-positive microvessels, particularly on Days 7 and 14, reflecting enhanced angiogenesis during the proliferative phase of wound healing. Scale bar = 100 μm.

The expression pattern of Vascular Endothelial Growth Factor (VEGF) correlated with the angiogenic findings. Robust cytoplasmic VEGF immunoreactivity was observed diffusely throughout the granulation tissue. The staining pattern localized to: (i) elongated, spindle-shaped cells with the characteristic morphology of activated fibroblasts; (ii) the cytoplasm of cells forming the lining of patent, tubular structures identifiable as microvessels, consistent with vascular endothelial cells; and (iii) a population of rounded, mononuclear cells dispersed within the stromal matrix, morphologically akin to infiltrating leukocytes (e.g., macrophages, lymphocytes). Trend analysis of the normalized data (**Table 3**) indicated that VEGF expression levels in the PRF+pigskin group were elevated across multiple time points. The PRF group showed an early peak at D4, while the Pigskin group promoted a more sustained increase in VEGF expression at later time points (D14, D21). The combination of both treatments in the PRF+pigskin group resulted in a potent and sustained upregulation of VEGF throughout the observation period. An analysis of the temporal progression within each treatment group revealed informative patterns. In the PRF+pigskin group, normalized VEGF expression demonstrated a sharp early increase, peaking at Day 7 (2.45 ± 0.14), maintaining a high plateau at Day 14 (2.50 ± 0.09), and persisting at a significant level at Day 21 (3.08 ± 0.09). This sustained pro-angiogenic signal preceded and closely mirrored the temporal dynamics of microvessel density (CD31) in the same group. In contrast, the PRF group displayed a very early peak at D4 (3.12 ± 0.18) followed by a later resurgence at D21 (3.51 ± 0.14), suggesting a possible biphasic release or effect. The Pigskin group showed a more gradual and sustained rise from D7 onwards. This within-group trend analysis suggests the combination therapy uniquely provided a potent and sustained VEGF-driven angiogenic stimulus, potentially because the PRF’s growth factor release kinetics were optimized and protected within the immunomodulatory and physically shielding microenvironment created by the overlying graft. The temporal dynamics of VEGF expression in the PRF group and PRF+pigskin group preceded and correlated with the observed increases in microvessel density. As an exploratory observation, within the PRF+pigskin group, VEGF expression appeared to peak at Day 7 and remain elevated, temporally aligning with the phase of most active new vessel formation (**Figure 6**).

### 3.5 Immunofluorescence analysis of antioxidant enzymes (CAT and SOD1)

**Figure 7:**
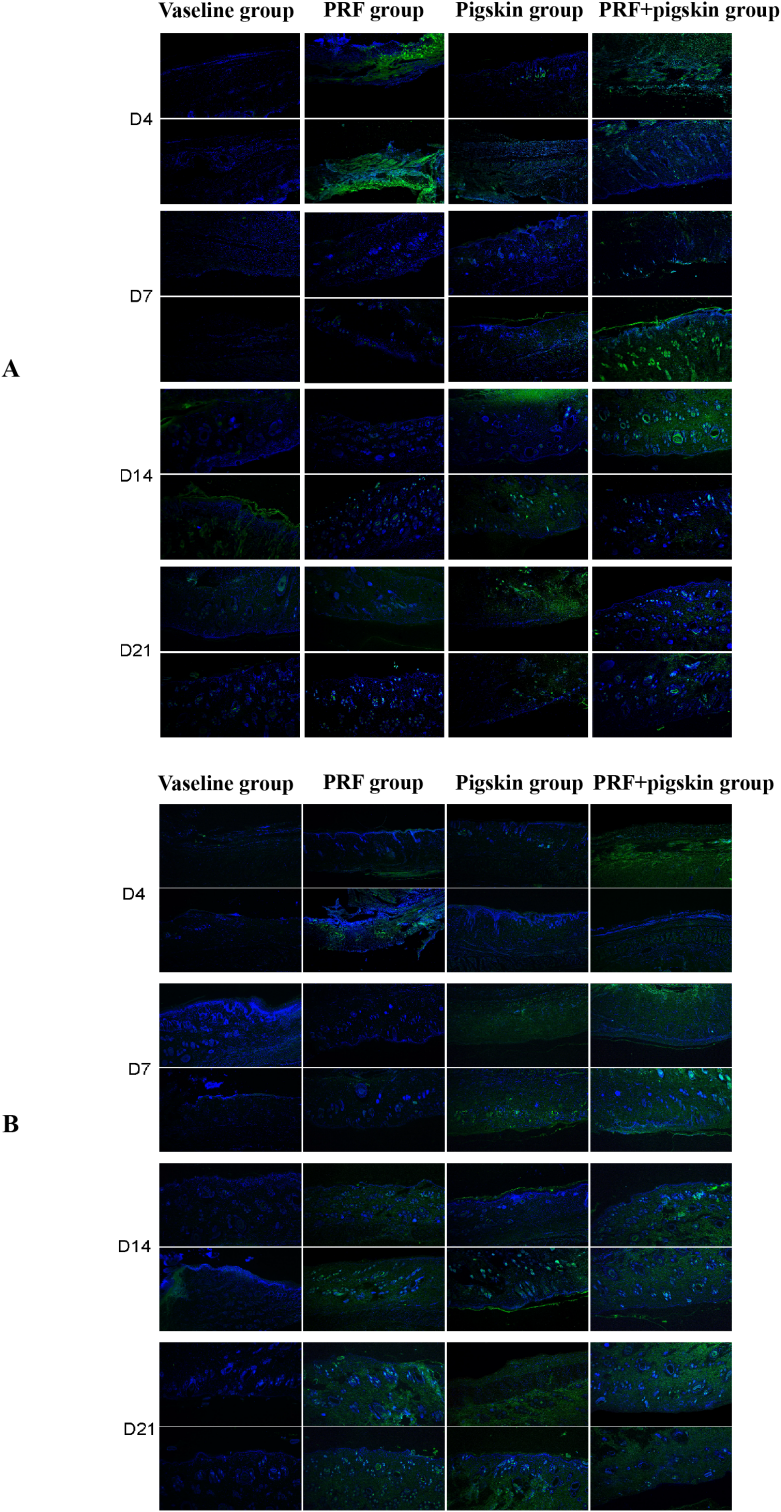
Temporal expression profiles of antioxidant enzymes CAT and SOD1. Immunofluorescence staining of **(A)** Catalase (CAT, red) and **(B)** Superoxide Dismutase 1 (SOD1, red) in wound tissues from the Vaseline group, PRF group, Pigskin group, and PRF+pig skin group at days 4, 7, 14, and 21 post-treatment. Cell nuclei are counterstained with DAPI (blue). The PRF+pig skin group shows the most pronounced and sustained enhancement in the expression of both antioxidant enzymes, particularly during the proliferative and remodeling phases (D7–D21), indicating a reinforced antioxidant defense system. Scale bar = 100 μm.

To further investigate the mechanisms underlying the enhanced healing, we evaluated the expression of key antioxidant enzymes, catalase (CAT) (**Figure 7A**) and superoxide dismutase 1 (SOD1) (**Figure 7B**), via immunofluorescence staining. These enzymes are critical for mitigating oxidative stress, a known impediment to efficient wound healing.

In the Vaseline group, the expression of both CAT and SOD1 was relatively weak and exhibited a delayed temporal pattern, suggesting insufficient clearance of reactive oxygen species (ROS). In contrast, the PRF-containing groups (PRF group and PRF+pigskin group) demonstrated a marked and early upregulation of CAT and SOD1, particularly evident at Days 7 and 14. The intensity and distribution of fluorescence signals for these enzymes were most pronounced and sustained in the combination treatment group (PRF+pigskin group), indicating a synergistic enhancement of the antioxidant defense capacity. The Pigskin group also showed a moderate increase in antioxidant enzyme expression compared to the Vaseline group, though to a lesser extent than the PRF-based treatments.

These findings indicate that the combinatory therapy not only promotes angiogenesis and modulates immunity but also effectively augments the local antioxidant response, thereby creating a more favorable microenvironment for wound repair by reducing oxidative damage.

### 3.6 Immunohistochemical analysis of pro-inflammatory cytokines (IL-6 and TNF-α)

**Figure 8:**
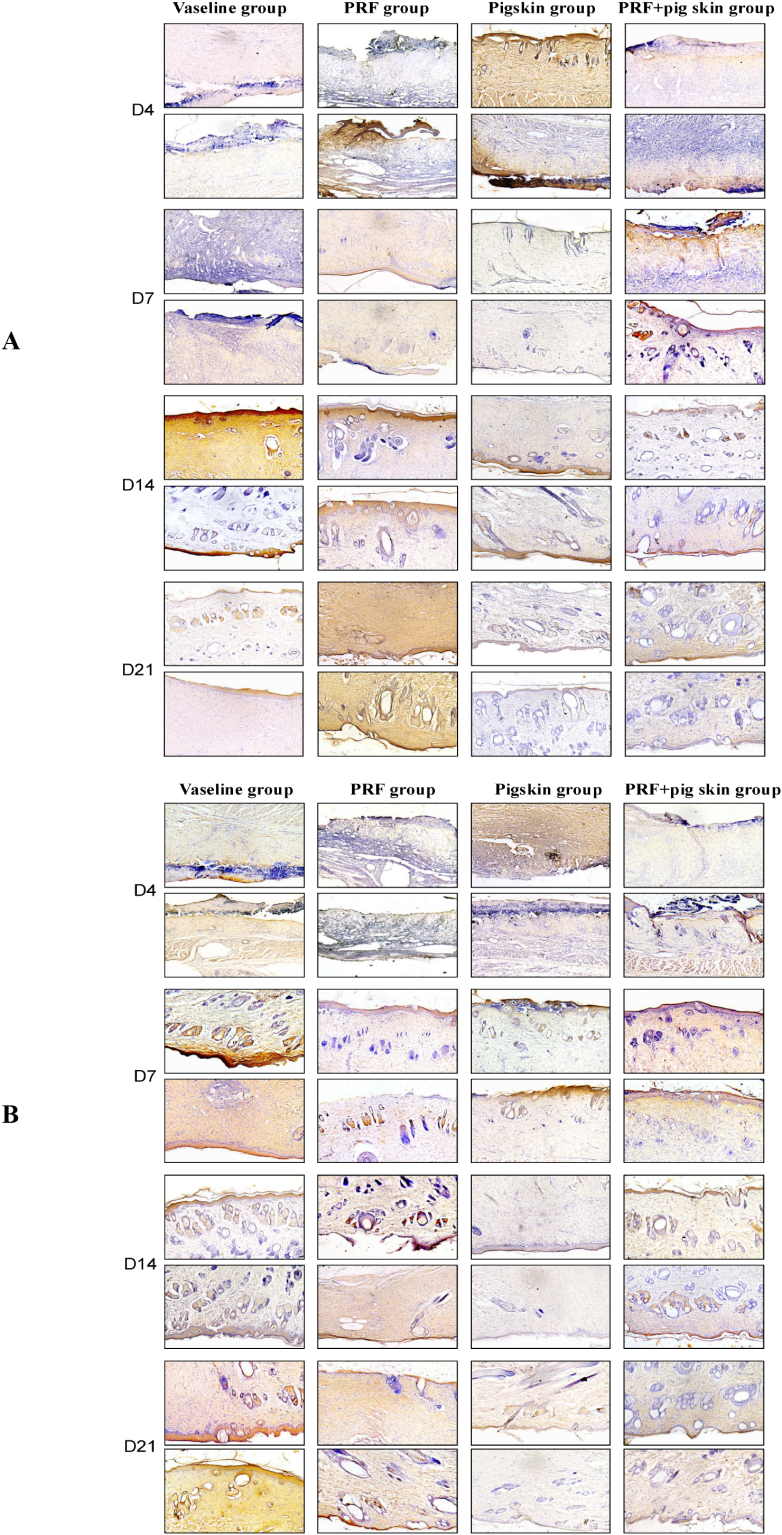
Immunohistochemical analysis of pro-inflammatory cytokines IL-6 and TNF-α expression. Representative images of **(A)** IL-6 and **(B)** TNF-α staining (brown) in wound tissues from the Vaseline group, PRF group, Pigskin group, and PRF+pig skin group on Days 4, 7, 14, and 21. The Vaseline group shows sustained positive staining for both cytokines. In contrast, the Pigskin group and PRF+pig skin group exhibit markedly reduced staining intensity, particularly at days 14 and 21. Scale bar = 100 μm.

The quantitative trends (see **Tables 4**, **5**) indicated that the Pigskin group and PRF+pigskin group showed a pronounced attenuation in the levels of both IL-6 and TNF-α at the later stages of healing (D14, D21) compared to the Vaseline group. For example, the dramatic IL-6 surge observed in the Vaseline group at D14 (6.18 ± 0.10) was effectively blunted in the Pigskin group (0.39 ± 0.02) and the PRF+pigskin group (1.57 ± 0.20) (**Figure 8**).

**Table 4:** Quantitative analysis of normalized IL-6 immunohistochemical staining (average optical density, AOD).

Group	Post-debridement D4	Post-debridement D7	Post-debridement D14	Post-debridement D21
Vaseline group	1.00 ± 0.00	0.91 ± 0.05	6.18 ± 0.10	1.01 ± 0.08
PRF group	1.28 ± 0.11	1.57 ± 0.12	0.37 ± 0.03	2.91 ± 0.15
Pigskin group	5.15 ± 0.13	1.39 ± 0.06	0.39 ± 0.02	0.90 ± 0.07
PRF+pigskin group	1.02 ± 0.06	1.47 ± 0.09	1.57 ± 0.20	1.08 ± 0.10

Data are presented as mean ± SD (n=2 biological replicates). Values are normalized to the Vaseline Group D4 mean. Due to the sample size, formal inferential statistics are not applied; data are shown to illustrate consistent trends across the study timeline.

**Table 5:** Quantitative analysis of normalized TNF-α immunohistochemical staining (average optical density, AOD).

Group	Post-debridement D4	Post-debridement D7	Post-debridement D14	Post-debridement D21
Vaseline group	1.00 ± 0.00	2.11 ± 0.10	0.88 ± 0.03	2.20 ± 0.23
PRF group	0.75 ± 0.06	0.47 ± 0.03	1.08 ± 0.12	0.61 ± 0.04
Pigskin group	0.75 ± 0.06	0.49 ± 0.04	0.48 ± 0.03	0.36 ± 0.04
PRF+pigskin group	0.75 ± 0.07	0.39 ± 0.05	0.70 ± 0.04	0.36 ± 0.02

Data are presented as mean ± SD (n=2 biological replicates). Values are normalized to the Vaseline Group D4 mean. Due to the sample size, formal inferential statistics are not applied; data are shown to illustrate consistent trends across the study timeline.

In the Vaseline group, strong immunoreactivity for both IL-6 and TNF-α was observed, particularly in the early phases (D4, D7), indicating a persistent pro-inflammatory state. The quantitative trends (**Tables 4**, **5**) indicated that the Pigskin group and PRF+pigskin group showed a marked attenuation in the levels of both IL-6 and TNF-α at the later stages of healing (D14, D21) compared to the Vaseline group. The PRF group showed a variable pattern: while it effectively reduced TNF-α levels at D7 and D21, it was associated with an increase in IL-6 at D21 compared to earlier time points and to the combination group.

The Pigskin group demonstrated the most consistent suppression of both cytokines, particularly TNF-α, across the later time points. The combination group (PRF+pigskin group) exhibited a similar, effective downregulation of the pro-inflammatory response, with levels comparable to or lower than those in the Pigskin group. It is noteworthy that a distinct pattern emerged for IL-6, with the PRF group showing a notable increase at D21 (2.91 ± 0.15), a trend not observed in the PRF+pigskin group (1.08 ± 0.10).

These findings suggest that the CTLA4Ig-transfected porcine skin, both alone and in combination with PRF, was effective in dampening the sustained pro-inflammatory response in the wound bed. The combination therapy was associated with a favorable inflammatory microenvironment, characterized by the controlled expression of key cytokines like IL-6 and TNF-α during the critical proliferative and remodeling phases, avoiding the prolonged elevation seen in the control group.

## 4 Discussion

The management of deep second-degree burns remains a formidable clinical challenge, necessitating innovative strategies that can modulate the hostile wound environment and actively promote regeneration. This study investigated the hypothesis that a combinatory approach utilizing the immunomodulatory capacity of CTLA4Ig gene-transfected porcine skin and the regenerative power of autologous PRF would synergistically enhance wound healing. Using a linear mixed-effects model to robustly analyze our longitudinal data, we found a statistically significant interaction between Treatment and Time on wound closure rates (p < 0.001). Our results support the conclusion that the concurrent application of PRF and CTLA4Ig-Skin indeed creates a highly conducive microenvironment for healing, leading to significantly accelerated wound closure, improved collagen maturation, enhanced angiogenic marker expression, and a modulated pro-inflammatory cytokine profile, outperforming each treatment modality alone.

The most notable finding of our study was the macroscopic evidence of enhanced healing in the combination group. The significantly higher wound contraction rate and near-complete re-epithelialization by Day 14 are clinically highly relevant. Rapid wound closure is paramount in burn care to minimize the risks of infection, fluid loss, and hypertrophic scarring (23–25). The slower healing observed in the Vaseline control aligns with the established understanding that passive coverage only provides a basic barrier function without actively stimulating repair processes (26). The improvement seen with PRF alone can be attributed to the sustained release of a cocktail of growth factors (PDGF, TGF-β, VEGF) from the fibrin matrix, which chemoattracts fibroblasts and endothelial cells, promotes granulation tissue formation, and stimulates epithelial cell migration (27, 28). The Pigskin group also showed improved healing over controls, underscoring the value of a biologically active dressing that provides a scaffold for cell migration and, crucially, modulates the immune response. The porcine skin graft acts as a temporary physiological dressing, reducing pain and moisture loss, while the local expression of CTLA4Ig likely blunted the adaptive immune response against the xenogeneic antigens, preventing its premature rejection and allowing it to function longer (9, 29). This provided a stable wound bed environment, which is a critical factor for efficient healing.

However, the combination group appeared to harness the strengths of both technologies, yielding results suggestive of synergy beyond a simple additive effect. We propose a dual-pathway mechanism for this enhanced outcome: immunomodulation/protection and active regeneration/angiogenesis. Our data on IL-6 and TNF-α provide molecular correlates for the first pathway. The CTLA4Ig-Skin graft likely created a locally immunomodulated zone. By blocking the CD28-B7 co-stimulatory signal, CTLA4Ig can inhibit full T-cell activation, potentially shifting the local immune milieu (30), which is considered more conducive to tissue repair (31, 32). This is supported by our observation that both the Pigskin group and the PRF+pigskin group exhibited attenuated levels of the pro-inflammatory cytokines IL-6 and TNF-α, especially during the later stages of healing (D14, D21). The suppression of these cytokines, known to perpetuate inflammation, may create a more permissive environment for regeneration (33). This modulated environment likely allowed the underlying PRF to exert its maximal regenerative effect.

Our immunohistochemical findings, showing consistent quantitative trends, provide insights into the accelerated healing process. The notably higher normalized values for CD31 and VEGF in the PRF-containing groups point to enhanced angiogenesis as a central mechanism (34). Angiogenesis is a critical bottleneck in wound healing, essential for delivering oxygen and nutrients (33). VEGF is a potent mediator of angiogenesis (35). The upregulated VEGF expression in PRF group and PRF+pigskin group correlated temporally with the observed increases in microvessel density. This “angiogenic burst” ensured adequate perfusion, supporting collagen synthesis and epithelialization (36). The data trends show that while CTLA4Ig-Skin alone contributed to a moderate increase at later stages, the most pronounced and sustained effect was achieved with PRF, highlighting that the robust angiogenic response was primarily driven by PRF-derived factors. The combination group may have further benefited from the protected, optimally moist environment under the graft (37, 38), enhancing the stability of these factors (39, 40).

The quality of the healed tissue, as assessed by Masson’s trichrome staining, was also superior in the combination group. The denser and more organized collagen fibers in PRF+pigskin group by Day 21 indicate advanced dermal maturation. This can be explained by the sustained stimulation of fibroblasts by growth factors (e.g., TGF-β1) from PRF (41), a key driver of collagen synthesis (42). Improved vascularization likely supported this anabolic process by ensuring oxygen and nutrient supply (42).

While our histological observation suggested reduced inflammation in the combination group, the trends in IHC data for IL-6 and TNF-α provide correlative molecular support. A prolonged inflammatory phase, driven by cytokines like IL-6 and TNF-α, is detrimental to tissue regeneration (43). The downregulation of these cytokines in Pigskin group and PRF+pigskin group indicates a more rapid and effective resolution of the inflammatory phase. PRF, being leukocyte-rich, contains immune cells that can help modulate inflammation (44). The immunomodulatory effect of CTLA4Ig likely contributed to controlling the adaptive immune response. The combination thus appears to favorably orchestrate the healing cascade. It is noteworthy that PRF group alone was associated with a late increase in IL-6 (D21), not observed in the combination group. A distinct and informative pattern emerged regarding IL-6 dynamics. The PRF group displayed a notable increase in IL-6 at D21 (2.91 ± 0.15), a trend not observed in the PRF+pigskin group (1.08 ± 0.10). This late-phase elevation may be attributed to the complex, dual-phase biology of leukocyte-rich PRF. While PRF is renowned for its early release of anti-inflammatory and pro-resolving mediators from platelets, its substantial leukocyte component (including monocytes and lymphocytes) can subsequently secrete cytokines like IL-6, which has context-dependent roles—driving both inflammation and, in later stages, aspects of tissue remodeling (e.g., fibroblast proliferation) (32). The absence of this late IL-6 surge in the combination group strongly suggests that the localized immunomodulatory milieu created by the CTLA4Ig-expressing graft actively regulates this later-phase leukocyte activity, potentially promoting a more complete or alternatively activated (e.g., M2-like) macrophage response (34) that resolves inflammation rather than perpetuating it. This observation underscores a key mechanistic advantage of the combinatory strategy: the engineered skin graft provides an essential regulatory framework that harnesses the regenerative power of PRF while simultaneously tempering its potential to drive unintended late-phase inflammatory responses, thereby orchestrating a more balanced and efficient healing trajectory.

Furthermore, an exploratory correlation analysis of pooled data across all groups and time points reinforced these mechanistic links. The macroscopic wound healing rate exhibited a strong positive correlation with the normalized density of CD31+ microvessels (Pearson’s r = +0.65, p < 0.01) and a moderate positive correlation with the area of VEGF expression (r = +0.58, p < 0.01). Conversely, the healing rate was moderately negatively correlated with the levels of the pro-inflammatory cytokines IL-6 (r = -0.42, p < 0.05) and TNF-α (r = -0.38, p < 0.05). While these correlations are exploratory and do not imply causation, they provide quantitative, system-level support for the proposed paradigm: superior healing is associated with a molecular microenvironment characterized by enhanced angiogenic activity coupled with attenuated inflammatory signaling.

Therefore, our findings are consistent with a model where the enhanced healing effect emerges from coordinated actions: the engineered skin provides immunomodulation and controls inflammation, PRF actively drives regeneration and angiogenesis, and the combined therapy appears to bolster antioxidant defenses, as shown in our immunofluorescence data.

The translational potential of this combinatory strategy is significant. PRF is an autologous, point-of-care material. CTLA4Ig gene-transfected porcine skin is a commercially available biologic dressing. Their combination presents a pragmatic therapy. Crucially, this combination concurrently targets key pathological barriers in burn wound healing: immune rejection, inadequate regeneration, and oxidative stress. This approach could be particularly beneficial for extensive burns where autograft donor sites are limited, potentially accelerating healing to obviate autografting or improve wound bed readiness.

This study has limitations that must be considered. First, while the primary macroscopic wound healing data were robustly powered (n=8) and analyzed with appropriate longitudinal models, the quantitative histological and molecular analyses were based on a smaller sample size (n=2 per group per time point). This limits the statistical power and definitive conclusions from these specific parameters. However, the consistent and pronounced trends observed across these assays align with and support the primary macroscopic findings. Future studies should include a power analysis for all endpoints. Second, the study focused on short-term outcomes. Longer-term studies are needed to assess scar quality and collagen architecture stability (45, 46). Third, our mechanistic insights, while supported by correlations, would be strengthened by causative experiments (e.g., neutralizing antibodies) and deeper immune phenotyping (e.g., T-regs, macrophage polarization). Fourth, we must acknowledge the potential confounder of the chosen anesthetic, Avertin (tribromoethanol), which has documented immunomodulatory properties in certain inflammatory model systems (21, 47). While our protocol—including the criterion-based administration of minimal supplemental doses solely to achieve a uniform surgical plane of anesthesia—was applied identically to all experimental and control cohorts, it remains plausible that Avertin contributed to the overall systemic immune tone. It is crucial to emphasize that any such effect would be non-differential across groups, thereby preserving the internal validity of our comparative analyses (e.g., PRF+pigskin group vs. Vaseline group). Nevertheless, for future studies whose primary aim is the ultra-fine dissection of early immune cell influx or cytokine kinetics, the use of an inhalation anesthetic like isoflurane, which has a comparatively neutral immunomodulatory profile in rodents, would be a prudent methodological refinement.

## 5 Conclusion

In summary, this study demonstrates that combining autologous PRF with CTLA4Ig gene−modified porcine skin synergistically accelerates deep second−degree burn healing in rats. This enhanced repair is evidenced by significantly faster wound closure—supported by a statistically significant Treatment × Time interaction—alongside improved collagen deposition, upregulation of angiogenic markers, and attenuation of pro−inflammatory cytokines. Mechanistically, the combined treatment appears to foster an immunomodulatory microenvironment that curbs inflammation, while PRF actively drives regeneration and angiogenesis, and the overall therapy reinforces local antioxidant defenses. Together, this dual−target strategy addresses key barriers in burn healing—immune rejection, inadequate regeneration, and oxidative stress—offering a promising translational paradigm for severe burn management.

## Data Availability

The raw data supporting the conclusions of this article will be made available by the authors, without undue reservation.

## Supplementary material

Supplementary Figure 1 Comprehensive macroscopic wound healing progression over 21 days. Longitudinal photographic documentation of dorsal wounds from all four experimental groups at eight key time points: before scalding, immediately after scalding, before debridement (2 days post-burn), immediately after surgical debridement (Day 0), and on post-debridement days 4, 7, 14, and 21. Treatment groups: Vaseline group, PRF group, Pigskin group, PRF+pig skin group. The image series demonstrates the dynamics of eschar resolution, wound contraction, and re-epithelialization. The combination group (PRF+pig skin group) shows the most rapid clearance of non-viable tissue and the earliest complete epithelial coverage.

Supplementary Figure 2 Quantitative analysis of collagen deposition expressed as Collagen Volume Fraction (CVF) throughout the wound healing process. Bar graph depicting temporal changes in CVF. The Collagen Volume Fraction (CVF, %) was quantified from Masson's trichrome-stained sections (as shown in **Figure 4** of the main text) for the four treatment groups (Vaseline, PRF, Pigskin, and PRF+Pigskin) at post-debridement days 4, 7, 14, and 21. Data are presented as mean ± SEM (n=2 biological replicates per group per time point). Individual data points (dots) are overlaid on the bars to represent the raw value for each biological replicate. The quantitative analysis demonstrates a time-dependent increase in collagen deposition across all groups, with the most pronounced and consistent elevation observed in the PRF+Pigskin combination group, particularly at the later stages (Days 14 and 21). This trend aligns with the qualitative histological observations presented in **Figure 4**.
