# Supplementary figures and images for: Synergistic effects of platelet-rich fibrin and CTLA4Ig gene-transfected porcine skin on accelerating wound healing in a rat model of deep second-degree burns: a mechanistic study
Source: Front Immunol. 2026 Jan 19;16:1756818. doi: 10.3389/fimmu.2025.1756818 (PMC12861883; doi:10.3389/fimmu.2025.1756818)

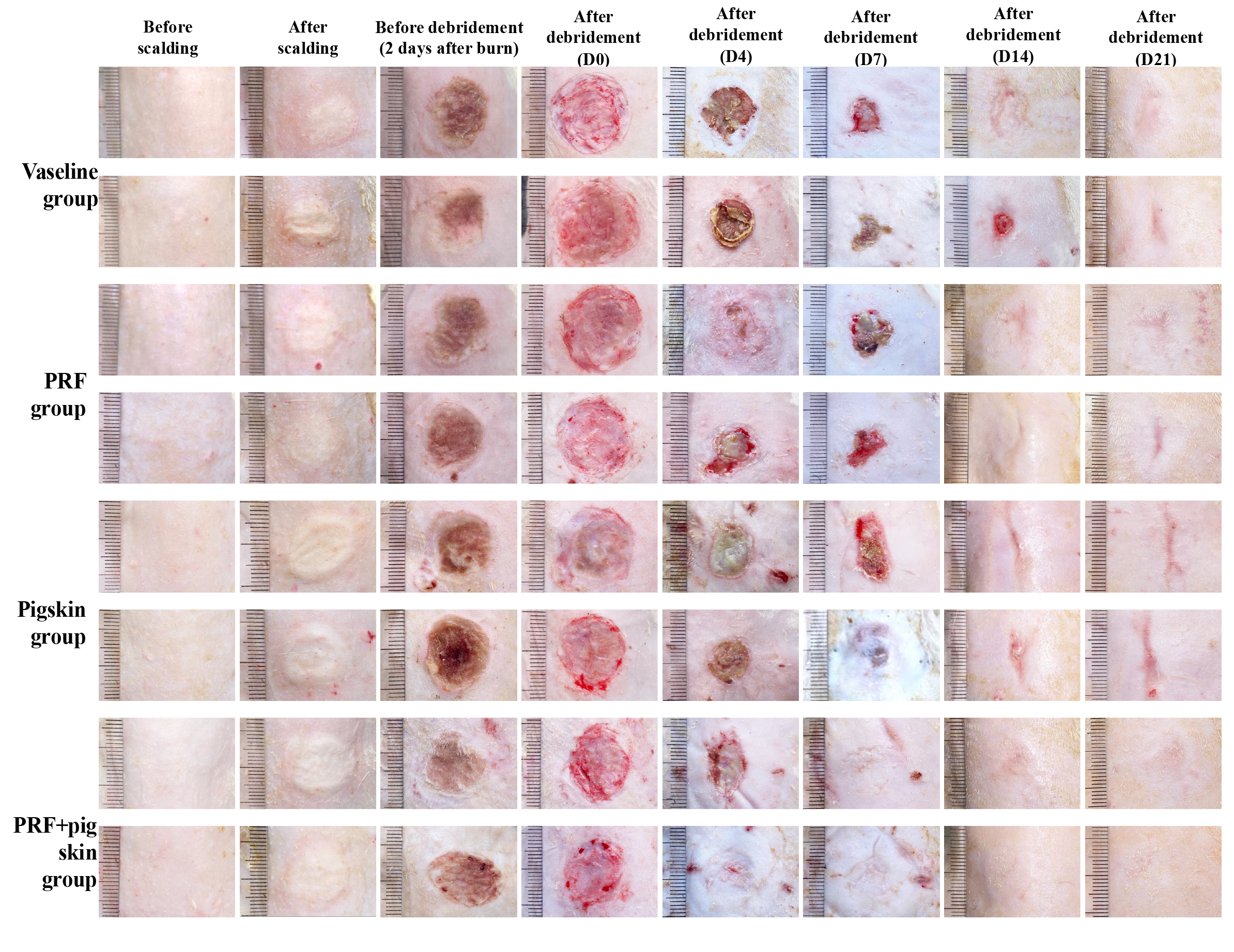

Supplement: Supplementary Figure 1 — Comprehensive macroscopic wound healing progression over 21 days. Longitudinal photographic documentation of dorsal wounds from all four experimental groups at eight key time points: before scalding, immediately after scalding, before debridement (2 days post-burn), immediately after surgical debridement (Day 0), and on post-debridement days 4, 7, 14, and 21. Treatment groups: Vaseline group, PRF group, Pigskin group, PRF+pig skin group. The image series demonstrates the dynamics of eschar resolution, wound contraction, and re-epithelialization. The combination group (PRF+pig skin group) shows the most rapid clearance of non-viable tissue and the earliest complete epithelial coverage. [file Image1.tif]

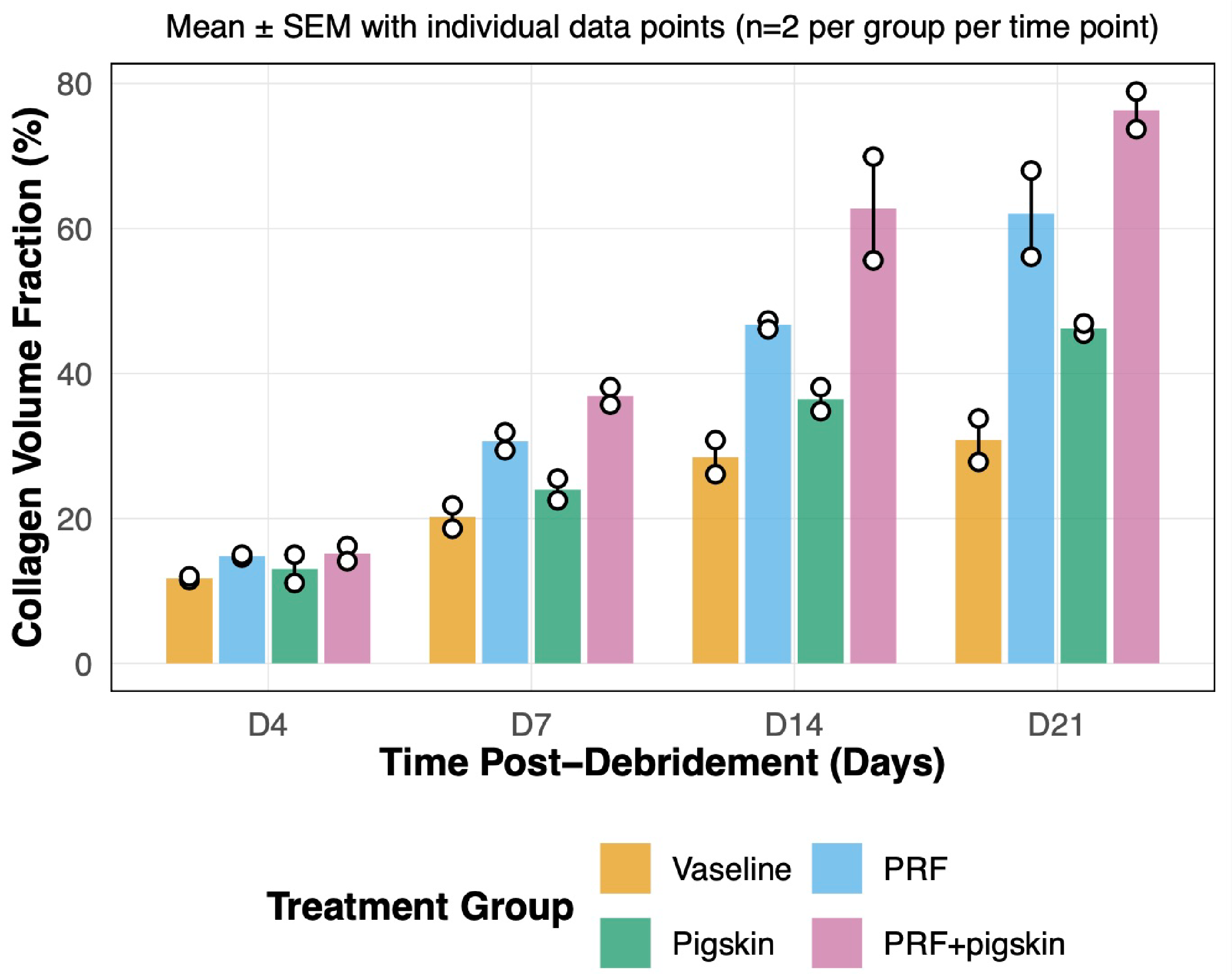

Supplement: Supplementary Figure 2 — Quantitative analysis of collagen deposition expressed as Collagen Volume Fraction (CVF) throughout the wound healing process. Bar graph depicting temporal changes in CVF. The Collagen Volume Fraction (CVF, %) was quantified from Masson's trichrome-stained sections (as shown in Figure 4 of the main text) for the four treatment groups (Vaseline, PRF, Pigskin, and PRF+Pigskin) at post-debridement days 4, 7, 14, and 21. Data are presented as mean ± SEM (n=2 biological replicates per group per time point). Individual data points (dots) are overlaid on the bars to represent the raw value for each biological replicate. The quantitative analysis demonstrates a time-dependent increase in collagen deposition across all groups, with the most pronounced and consistent elevation observed in the PRF+Pigskin combination group, particularly at the later stages (Days 14 and 21). This trend aligns with the qualitative histological observations presented in Figure 4 . [file Image2.tif]
