# Supplementary material for: Synergistic effects of platelet-rich fibrin and CTLA4Ig gene-transfected porcine skin on accelerating wound healing in a rat model of deep second-degree burns: a mechanistic study
Source: Front Immunol. 2026 Jan 19;16:1756818. doi: 10.3389/fimmu.2025.1756818 (PMC12861883; doi:10.3389/fimmu.2025.1756818)
Supplement: Supplementary file 3 [file Supplementaryfile1.docx]

| **Supplementary Table S1. Collagen Volume Fraction (CVF) in wound tissue at different time points** | | | | |
| --- | --- | --- | --- | --- |
| **Group** | **Post-debridement D4 (Mean ± SD)** | **Post-debridement D7 (Mean ± SD)** | **Post-debridement D14 (Mean ± SD)** | **Post-debridement D21 (Mean ± SD)** |
| **Vaseline group** | 12.3 ± 1.5 % | 18.5 ± 2.1 % | 25.7 ± 3.0 % | 32.2 ± 3.5 % |
| **PRF group** | 15.8 ± 1.8 % | 28.4 ± 2.9 % | 45.6 ± 4.2 % | 58.9 ± 5.1 % |
| **Pigskin group** | 14.2 ± 1.6 % | 23.7 ± 2.5 % | 38.9 ± 3.8 % | 50.3 ± 4.7 % |
| **PRF+pigskin group** | **17.5 ± 2.0 %** | **35.2 ± 3.5 %** | **62.4 ± 6.0 %** | **75.8 ± 7.2 %** |

**Note:** Data are presented as mean percentage of collagen-stained area relative to total tissue area ± standard deviation (SD) (n=2 biological replicates per group per time point). The CVF values illustrate the trend of collagen deposition over time, with the combination group (PRF+pigskin group) showing the most pronounced increase, especially at Days 14 and 21, consistent with the qualitative observations from Masson's trichrome staining. **These data are presented for illustrative trend analysis and were not subjected to formal inferential statistical testing due to sample size constraints.**
