# Supplementary material for: Synergistic effects of platelet-rich fibrin and CTLA4Ig gene-transfected porcine skin on accelerating wound healing in a rat model of deep second-degree burns: a mechanistic study
Source: Front Immunol. 2026 Jan 19;16:1756818. doi: 10.3389/fimmu.2025.1756818 (PMC12861883; doi:10.3389/fimmu.2025.1756818)
Supplement: Supplementary file 4 [file Supplementaryfile2.docx]

| **Supplementary Table S2. Original two-way ANOVA results for the effects of Treatment, Time, and their Interaction on Wound Healing Rate** | | | | | |
| --- | --- | --- | --- | --- | --- |
| **Source** | **Sum of Squares** | **df** | **Mean Square** | **F-value** | **p-value** |
| **Treatment** | 4.58 | 3 | 1.527 | 185.4 | **< 0.001** |
| **Time** | 9.69 | 3 | 3.230 | 392.7 | **< 0.001** |
| **Treatment × Time** | 2.14 | 9 | 0.238 | 28.9 | **< 0.001** |
| **Residual** | 0.92 | 112 | 0.008 |  |  |

**Note:** This two-way analysis of variance (ANOVA) was performed on the wound healing rate data, with Treatment and Time (Day 4, 7, 14, 21) as independent factors. The dependent variable was the wound healing rate (%). The analysis demonstrated a significant interactive effect of Treatment and Time on the healing rate (F(9, 112) = 28.9, p < 0.001). **This specific analysis, which initially indicated a significant Treatment × Time interaction (p < 0.001), was superseded by the more robust Linear Mixed-Effects Model reported in the main text (Table 1), which more appropriately accounts for repeated measures within subjects. It is presented here for completeness and to directly address the reviewer's query.**
